# Connexin45 colocalization patterns in the plexiform layers of the developing mouse retina

**DOI:** 10.1111/joa.13651

**Published:** 2022-03-21

**Authors:** Gerrit Hilgen

**Affiliations:** ^1^ Health & Life Sciences, Applied Sciences Northumbria University Newcastle upon Tyne UK; ^2^ Biosciences Institute Newcastle University Newcastle upon Tyne UK

**Keywords:** connexin, Cx36, Cx45, development, gap junction, retina, synaptogenesis

## Abstract

Chemical and electrical synapses (gap junctions) are widely prevalent in the nervous system. Gap junctions emerge long before chemical synapses, allowing communication between developing cells, and are thought to be involved in establishing neural circuits. Mounting evidence indicates that these two modalities of synaptic transmission closely interact during retinal development and that such interactions play a critical role in synaptogenesis and circuit formation during the perinatal period. In vertebrates, gap junctions consist of two connexins which in turn are made up of six connexins (Cx). To what extent Cx45 and Cx36, the most abundant connexins in the retina, are involved in synaptogenesis and retinal circuit formation is not known. The here presented immunohistochemical study used stainings of Cx45, Cx36 and Synaptophysin in the outer and inner (IPL) plexiform layers of postnatal day 8–16 mice retinas to shed light on the role of connexins during critical neuronal developmental processes. Cx45 and Cx36 expressions in both plexiform layers of the mouse retina increased till eye opening and dropped afterwards. The percentage of heterotypic Cx45/Cx36 gap junctions is also higher before the critical event of eye opening. Finally, Cx45 is closely located and/or colocalized with Synaptophysin also shortly before eye opening in the IPL of the mouse retina. All findings point towards a pivotal role for Cx45 during postnatal synaptogenesis in the mouse retina. However, a more functional study is needed to determine the role of Cx45 during synaptogenesis and circuit formation.

## Abstract 2

Cx45 expression and colocalization with Synaptophysin, a marker for presynaptic terminals and synaptogenesis, is stronger before eye opening in the mouse retina. Heterotypic Cx45/Cx36 gap junctions are also more frequent before the critical event of eye opening.

## 1 INTRODUCTION

Direct cell–cell connections, so‐called gap junctions, are present in all multicellular organisms and are critical for the organism’s integrity, metabolism and physiology (Evans & Martin, 2002). Gap junctions are widely prevalent in the central nervous system, allowing neurons to directly communicate with each other mediated by direct connection between adjacent neurons through two hemichannels (Alcamí & Pereda, 2019). In vertebrates, one gap junction consists of two connexons (hemichannels) and one connexon is made up of six cell membrane‐spanning proteins, the connexins (Cx). The relatively high sequence similarity between the ~20 members of the connexin family in mice and humans allows the comparison of functional and physiological aspects across these species (Evans & Martin, 2002; Söhl & Willecke, 2004).

The retina is the light‐sensitive tissue lining the back of the eye. It is a well‐layered structure with two synaptic layers, the outer (OPL) and inner (IPL) plexiform layers. Photoreceptors synapse to bipolar cell dendrites and horizontal cells in the OPL, whereas bipolar cell terminals synapse to amacrine and ganglion cells in the IPL (Masland, 2012). The two most abundant connexins in the mouse retina are Cx45 and Cx36 (Bloomfield & Volgyi, 2009; Sigulinsky et al., 2020). Cx45 is expressed in bipolar cells (Dedek et al., 2006; Hilgen et al., 2011; Maxeiner et al., 2005) and between ganglion cells (Schubert, Maxeiner, et al., 2005). Cx36 is expressed between cones (Jin et al., 2020; O'Brien et al., 2012), dendrites of bipolar cells (Feigenspan et al., 2004; Han & Massey, 2005), aII amacrine cells (Deans et al., 2002; Feigenspan et al., 2001), amacrine and ipRGC cells (Harrison et al., 2021) and between ganglion cells (Pan et al., 2010; Schubert, Degen, et al., 2005). Cx45 and Cx36 form heterotypic junctions; AII amacrine cells are coupled by Cx36 and transfer their signals via a Cx36/Cx45 gap junction to ON‐bipolar cells which constitutes an essential element of the primary rod pathway (Dedek et al., 2006; Wässle, 2004).

In the developing nervous system, gap junctions emerge first, long before chemical synapses, allowing communication between developing cells. At later stages, newly formed chemical synapses can lead to the elimination of these first gap junctions in most neuronal types (Jabeen & Thirumalai, 2018; Pereda, 2014). The physiological necessity of gap junctional communication during early development, and especially for synapse formation (synaptogenesis), is still controversially discussed. Synaptogenesis in the IPL and OPL of the developing mouse retina begins shortly before eye opening and continues for several weeks (Tian, 2004). Mice open their eyes around 2 weeks after birth (postnatal day [P] 12–14). This is a critical period for the retina as most synapses in both plexiform layers are further shaped (Fan et al., 2016; Lohmann & Kessels, 2014), ganglion cell dendritic stratification in the IPL matures (Tian, 2011) and light‐driven activity is further refined (Hilgen et al., 2017; Tian & Copenhagen, 2001, 2003). Before eye opening, Cx45 and Cx36 correlate the spontaneous activity of nearby ganglion cells and ensure thereby normal patterning of retinogeniculate projections during retinal development. Previous studies reported the developmental expression patterns of certain connexins in the rat retina (Cx36, Cx37, Cx43, Cx45, Cx50, Cx57; Kihara et al., 2006b; Kihara et al., 2010; Kovács‐Öller et al., 2014) around eye opening. However, little is known about the colocalization patterns of Cx45 with other proteins for the critical time period around eye opening. The here presented study aims to reveal the developmental expression pattern of Cx45 in the OPL and IPL of the mouse retina, as well as its colocalization with Cx36 and Synaptophysin, a marker for synapse forming (Fan et al., 2016; Kihara et al., 2008), around the time of eye opening. These results will extend the current knowledge of retinal Cx45 expression during retinal development as well as its interaction with Cx36 and synapse‐forming proteins.

## 2 METHODS

### 2.1 Animals and retina preparation

All experimental procedures were approved by the ethics committee at Newcastle University and carried out in accordance with the guidelines of the UK Home Office, under the control of the Animals (Scientific Procedures) Act 1986. Male and female wild‐type mice, housed under a 12‐h light‐dark cycle and aged between postnatal days (P) 8–16 were used for the experiments. Mice were killed by cervical dislocation. Eyes were enucleated, and following removal of the cornea, lens and vitreous body, they were placed in oxygenated 0.1 M phosphate buffer solution (PBS) (Sigma). The retina was isolated from the eyecup and flattened on nitrocellulose filter paper. Mouse eyecups were fixed in 4% paraformaldehyde (Alfa Aesar) in 0.1 M PBS for 30 min at room temperature and washed with PBS several times. Eyecups were cryoprotected in 30% sucrose in PBS overnight at 4°C and embedded in OCT Tissue TeK (Sakura) at −20°C on the following day. Vertical sections (15–20 μm) were cut on an OTF5000 cryostat (Bright Instruments) and collected on Superfrost microscope slides (Thermo Fisher).

### 2.2 Immunohistochemistry (IHC)

Mouse retinal and sections were blocked with 10% normal goat serum in PBS for at least 30 min at room temperature. After the blocking procedure and a short rinse in PBS, vertical sections were incubated with primary antibodies in 0.5% Triton X‐100 + PBS overnight at 4°C. Incubation with secondary antibodies in 0.5% Triton X‐100 in PBS was carried out for 2 h at room temperature for sections. Details of the primary antibodies are as follows: anti‐Cx45 (rabbit polyclonal, Connexin45 Cytoplasmic loop, aa 143–169, 1:500, kind gift U. Janssen‐Bienhold and K. Dedek, University of Oldenburg, Germany, Dedek et al., 2006), anti‐Cx36 (mouse monoclonal, Connexin36 C‐terminal region of rat, 1:1000, Thermo Fischer 374600) and anti‐synaptophysin (mouse monoclonal, MAB368, Millipore). All primary antibodies work specifically in mice and humans. Secondary antibodies are as follows (all concentrations 1:500): goat anti‐rabbit Atto594 and goat anti‐mouse Atto488. After washing several times with PBS, sections were mounted in ProLong Diamond with Hoechst 33342 (1:1000) for nuclei staining.

### 2.3 Image acquisition and processing

Images were captured using a Leica TCS SPE confocal microscope with 63× oil objectives (Zeiss) operated with LAS software and 405, 488, 532 and 635 nm solid‐state lasers for excitation. For retinal sections, 1024 × 1024 px stacks (0.5 μm z step) with the 63× objective and with 3× (for IPL) and 4× (for OPL) digital zoom were acquired. All image postprocessing was done with Fiji (https://fiji.sc), Adobe Photoshop (Adobe) and MATLAB (Mathworks). Briefly, the Leica Image Files were imported in Fiji and the different channels were converted to 8‐bit. Channel histogram levels were slightly adjusted (using a region of interest [ROI] and autofunction), the background subtracted (50 pixels) and outliers removed (threshold 0, pixel 2). Single sections from the beginning, middle and end of the stack were isolated. An ROI was drawn around the entire IPL or OPL, respectively, on these sections and a threshold (maximum entropy) was applied for each section and channel. The particle size threshold was set to 0.05–1 μm which proved to be very efficient to exclude the stripe patterns for analysis. For all thresholded particles, the density and average size were calculated. The threshold particles were imported (as a mask) in the image calculator and the ‘AND’ operator revealed the overlap between the two channels. For such colocalized particles, the density and average size were also calculated (for particles between 0.05 and 1 μm). All measured values were exported to Matlab and boxplots for the different ages were generated with BoxPlotPro (https://www.mathworks.com/matlabcentral/fileexchange/88733‐boxplotpro). Shapiro–Wilk test was used to test normality. An ANOVA with Tukey–Kramer post hoc test was used to estimate if the means of the groups significantly differ from each other.

## 3 RESULTS

**FIGURE 1:**
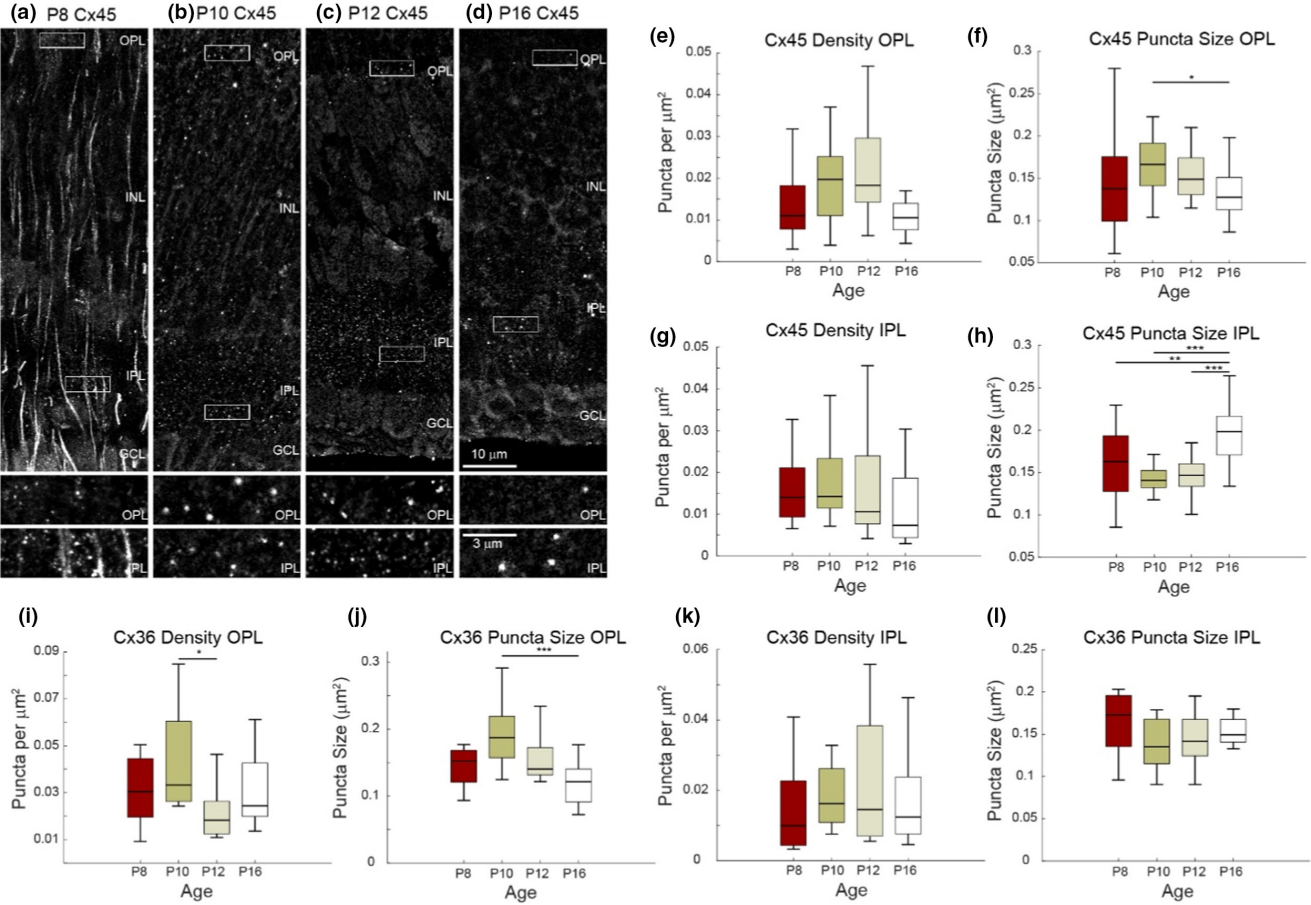
Quantification of Cx45 and Cx36 densities and puncta sizes in the plexiform layers of early postnatal retinas. Cx45 IHC expression patterns in single vertical sections (0.5 mm^2^ thick) of P8 (a), P10 (b), P12 (c) and P16 retinas (d). Insets show high magnifications from the OPL and IP, respectively. Boxplots (median + 25^th^/75^th^ percentile) are used to visualize the Cx45 densities (puncta/mm^2^) in the OPL (e) and IPL (g) as well as the puncta sizes (mm^2^) in the OPL (f) and IPL (h). In the same way, densities (i OPL; k IPL) and puncta sizes (j OPL; l IPL) were calculated for Cx36. The number (*n*) of analysed retinal single sections are given as follows: (e, f) P8 (60), P10 (72), P12 (54), P16 (66); (g, h): P8 (48), P10 (60), P12 (60), P16 (60); (i, j) P8 (24), P10 (30), P12 (24), P16 (36); (k, l) P8 (30), P10 (30), P12 (27), P16 (30). GCL, ganglion cell layer; INL, inner nuclear layer; IPL, inner plexiform layer; OPL, outer plexiform layer. *p <0.05, **p <0.01, ***p <0.001. Scale bars (a–d) top = 10 μm, bottom = 3 μm

To reveal the Cx45 immunoreactivity (IR) pattern for the developing retina, cryosections from P8, 10, 12 and 16 mouse retinas (Figure 1a–d) were stained with an established antibody against Cx45 (Dedek et al., 2006; Hilgen et al., 2011) followed by imaging high magnifications from the OPL and IPL. P8 showed some streaky cross‐labelling but also Cx45 puncta pattern (Figure 1a, insets) which the image processing settings were able to extract and analyse. The streaky pattern disappeared in P10, P12 and P16 (Figure 1b–d, insets) retinas and the plexiform layers had the typical punctate pattern of connexins. At a first glance, the Cx45 density (measured as puncta per μm^2^) and puncta size (measured as μm^2^) varied between different ages in both plexiform layers (Figure 1a–d bottom insets). To confirm this observation, the density and puncta size of Cx45 in single sections of the OPL and IPL were quantified (Figure 1e–h). The Cx45 density in the OPL does not significantly differ between the different ages (Figure 1e) although the highest median is observed at P10. In line with this finding, the puncta size in the OPL has its highest value also at P10 (Figure 1f). The difference between P10 and P16 puncta size was significant (*p* = 0.0374). Like in the OPL, a similar Cx45 density pattern was observed for the IPL, showing the highest Cx45 density around P10 and its lowest density at P16 (Figure 1g). In contrast to the puncta size in the OPL, the puncta size in the IPL significantly increased from P10 to its largest size at P16 (Figure 1h). The Cx45 median puncta sizes between P10, P12 and P8 do not significantly differ from each other, whereas the P16 puncta size significantly differs from all of them.

The other most abundant connexin in the retina is Cx36. The highest Cx36 median density in the OPL was found around P10 and the lowest around P12 (*p* = 0.0446) (Figure 1i). The largest Cx36 median puncta size in the OPL appeared at P10 while the smallest at P16 (*p* = 0.0007) (Figure 1j). The highest Cx36 median densities were found at P10 and P12 in the IPL while P18 and P16 showed less Cx36 puncta (Figure 1k). The IPL of P8 retinas consists of a higher Cx36 median puncta size with P16 slightly smaller) and P10/P12 the lowest (Figure 1l).

In summary, Cx45 had its peak density expression between P10 and P12 in both plexiform layers. The median size of the Cx45 gap junction plaque peaked also at P10 in the OPL in contrast to the IPL, where the greatest size was reached at P16. Cx36 had its peak density expression at P10 in both plexiform layers. The Cx36 puncta size was slightly higher in the OPL at P10 in contrast to the IPL, where P8 retinas had the largest Cx36 plaques.

**FIGURE 2:**
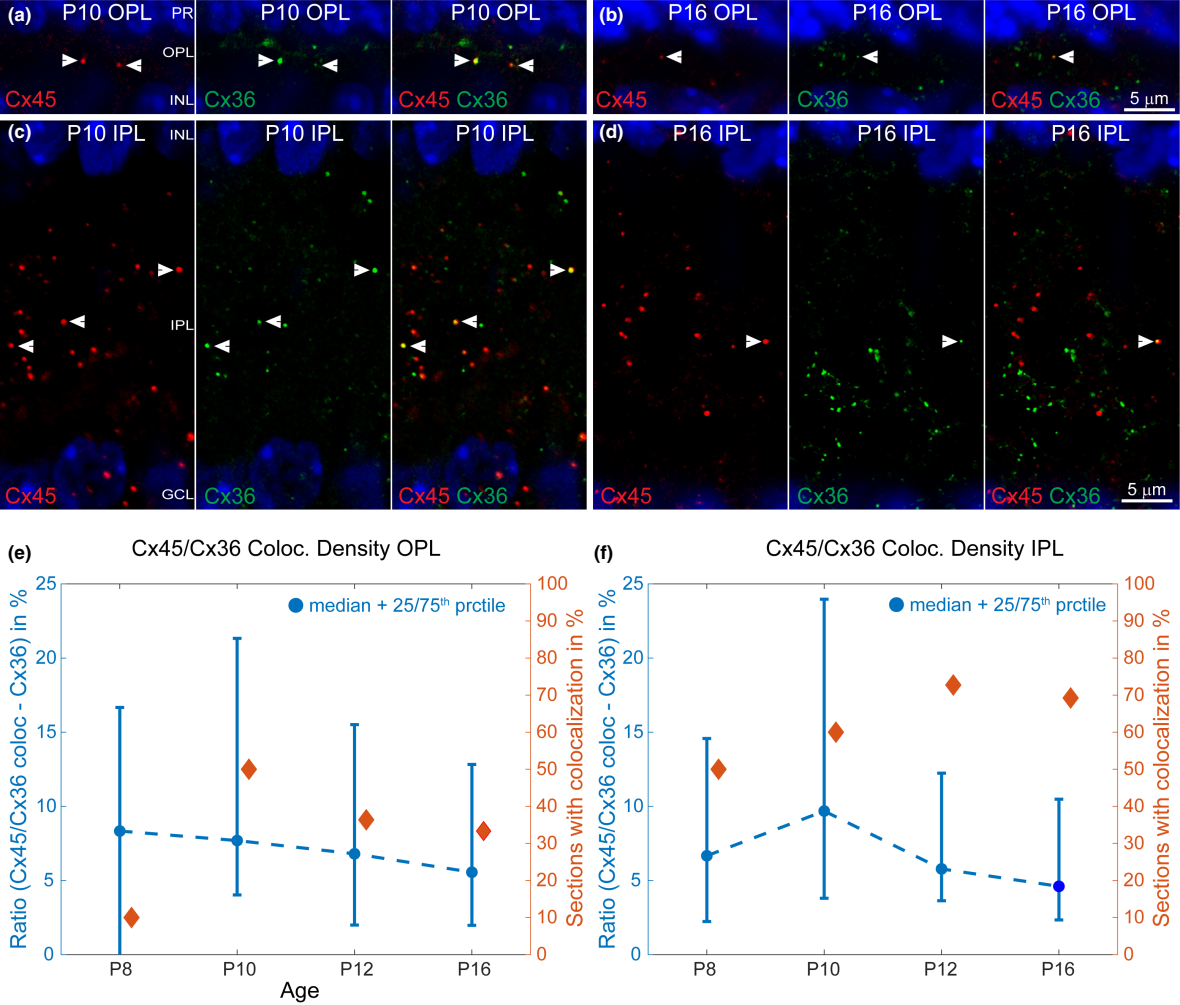
Cx45/Cx36 colocalizations before and after eye opening. Magnifications of representative Cx45/Cx36 colocalizations in single sections of a P10 and a P16 retina (a–d). Cx45/Cx36 colocalizations are more numerous before eye opening in the OPL (a) and IPL (c) compared to similar areas in the OPL (b) and IPL (d) after eye opening. Median ± 25^th^/75^th^ percentile of the ratio of Cx45/Cx36 colocalization densities are plotted on left *y* axis against age on the *x* axis (e, f, blue). The percentage of single sections that showed a Cx45/Cx36 colocalization is plotted against the right *y* axis (e, f, red). Both parameters help to visualize the developmental pattern of Cx45/Cx36 heterotypic gap junctions in the OPL (e) and the IPL (f). The number (*n*) of analysed retinal single sections are as follows: (e): P8 (27), P10 (27), P12 (33), P16 (39); (f): P8 (33), P10 (27), P12 (33), P16 (42). Scale bars in (a–d) are 5 μm. IPL, inner plexiform layer; OPL, outer plexiform layer

Both connexins often form heterotypic gap junctions in the IPL as part of the primary rod pathway. To answer how often these two connexins form heterotypic junctions during the postanal refinement period of the retina, single sections of the OPL and IPL were analysed for potential differences between Cx45/Cx36 expression patterns throughout the development. More Cx45/Cx36 gap junctions were observed before eye opening (Figure 2a,c, arrowheads) than after eye opening (Figure 2b,d, arrowheads) in the OPL (Figure 2a,b) and IPL (Figure 2c,d). However, most of the analysed single sections did not show a colocalization and the focus was on the ones that did show a colocalization (Figure 2e,f). The percentage of sections that did show a Cx45/Cx36 colocalization was highest at P10 in the OPL (Figure 2e, right axis, red) and P12 in the IPL (Figure 2f, right axis, red). The ratio (in %) of C45/Cx36 gap junction densities in relation to all Cx36 gap junction densities were in both plexiform layers higher before P12 (eye opening). In the OPL, the highest Cx45/Cx36 colocalization value belonged to P8 retinas (Figure 2e, left axis, blue), whereas for the IPL, it was P10 (Figure 2f, left axis, blue). However, the statistical test could not find any significant differences. . In summary, Cx45/Cx36 gap junctions are more likely before eye opening in both plexiform layers and their density is slightly higher at P8/P10 respectively.

**FIGURE 3:**
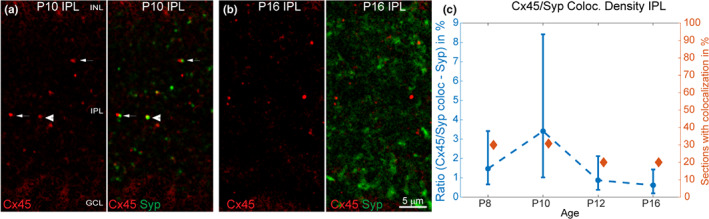
Cx45/Syp colocalizations in developing mice retinas. Magnifications of representative Cx45/Syp colocalizations in single sections of a P10 (a) and a P16 (b) mice retinas. Cx45/Syp colocalizations are more numerous before eye opening in the IPL (a, b). (c) Median ± 25^th^/75^th^ percentile values of Cx45/Syp colocalization density ratios (in %) are plotted on the left *y* axis (blue) against age on the *x* axis to visualize the developmental pattern of Cx45/Syp structures in the developing IPL. Additionally, the percentages of single sections with colocalizations are plotted on the right *y* axis (red) against age on the *x* axis. The number (*n*) of analysed retinal single sections are as follows: (c): P8 (27), P10 (39), P12 (33), P16 (27). Scale bar in (b) is 5 μm. IPL, inner plexiform layer

Synaptophysin (Syp) is a marker for presynaptic terminals and is here used to investigate synaptogenesis in the OPL and IPL. Before eye opening, Syp showed a small punctate pattern (Figure 3a, green) and later it became more pronounced and synapse‐like shaped (Figure 3b, green) in the IPL, potentially reflecting the bipolar synapses. It is also localized on the photoreceptor terminal in the proximal OPL but not in the distal OPL. As Cx45 is not expressed in photoreceptors (Jin et al., 2020), no colocalizations of Syp with Cx45 in the proximal OPL was found, hence the focus was only on the IPL. Two patterns emerged: Cx45 is localized close to (Figure 3a, arrow) or on (Figure 3a, arrowhead) forming synapses in the IPL during postnatal retinal development. Cx45 showed by eye visible higher colocalizations with Syp before eye opening (Figure 3a,b, arrowheads) in the IPL. The ratio (in %) of colocalized Cx45/Syp structures against all Syp structures was calculated (Figure 3c, left axis, blue) and confirmed a higher percentage of Cx45/Syp densities at P10. Additionally, the percentages of single sections with Cx45/Syp colocalizations were calculated (Figure 3, right axis, red) and did show slightly higher values at P8 and P10 than at later ages. NB the differences between all values were not significant. In summary, Cx45 interacts potentially more with Syp before eye opening than afterwards.

## 4 DISCUSSION

This study shows that Cx45 expression in both plexiform layers of the mouse retina is stronger before eye opening. The Cx45 puncta size develops differently; larger Cx45 gap junctions can be found before eye opening in the OPL, whereas the IPL shows the largest Cx45 gap junction after eye opening. Heterotypic gap junctions with Cx45 and Cx36, which can be often found in the primary rod pathway, are also more frequent before the critical event of eye opening in both layers. Cx45 is closely located and/or colocalized with Synaptophysin, which is a marker for presynaptic terminals and synaptogenesis in the retina (Fan et al., 2016; Kihara et al., 2008), shortly before eye opening in the IPL of the mouse retina.

The P8 retinas showed streaky vertical structures in the Cx45 channel (Figure 1a), hence the image processing was adjusted to solely extract punctate Cx45 pattern. These streaky structures could potentially reflect the intermediate filament protein vimentin (Kihara et al., 2006b; Lemmon & Rieser, 1983) which is expressed by Mueller cells. It could also resemble cytosolic Cx45 expression in Mueller cells. But Cx45 expression in Mueller cells is only reported for the rat retina (Kihara 2006; Zahs et al., 2003) but not for the mouse retina. However, the streaky staining is no anymore present in >P10 retinas and its expression changes to puncta, typical for connexin IR (Figure 1b–d). A previous study reported that Cx45 steadily decreases from P1 to P60, whereas the here presented results rather suggest a peak at P10 followed by a steadily decrease (Kihara et al., 2006b). Kihara et al. (2006b) quantified the Cx45 mRNA in the rat retina, whereas in this study, the Cx45 protein expression in the IPL and OPL of the mouse retina was quantified using IHC. However, the principal development pattern of Cx45 – more Cx45 expression before eye opening – is comparable to Kihara et al. (2006b). NB Connexins are not homogenously distributed across the retina (Fusz et al., 2021) and their expression can be modulated (Chen et al., 2021; Janssen‐Bienhold et al., 2009; Kihara et al., 2006a). For the analysis, we used cryosections from the same areas and preparations/preparation time and pre‐dark adaptations were the same for all ages and mice.

More Cx45/Cx36 heterotypic gap junctions were found before eye opening, which could be correlated to the high expression of Cx45 and Cx36 at that time. The observed Cx36 density in the IPL is also in line with previous studies (Kihara et al., 2006b; Kovács‐Öller et al., 2014). The latter study looked at the Cx36 puncta size which was comparable to the here presented results in the IPL. Heterotypic Cx36/Cx45 gap junctions can be found in the OFF and ON layer of the IPL (Dedek et al., 2006) of the adult mouse retina. In contrast to adult retinas, most of the Cx45/Cx36 junctions in the IPL, were observed in the OPL at P10. If and how these Cx45/Cx36 gap junctions in the IPL relate to the AII amacrine cell – ON‐bipolar cell junction (primary rod pathway) needs to be seen. It could be possible that Cx45 and Cx36 play a pivotal role in AII amacrine circuit development. There is no specific whole‐cell marker for AII amacrine cells in mice retinas available and AII and bipolar cells have to be cell filled to investigate that junction during development (Meyer et al., 2014) in a future study. The Cx45/Cx36 gap junctions in the OPL can only arise from bipolar cell dendrites as Cx45 is not in mouse photoreceptors and horizontal cells (Dedek et al., 2006; Hilgen et al., 2011; Janssen‐Bienhold et al., 2009; Jin et al., 2020), at least not in adult retinas. Again, both connexins could play a role in OPL circuit development and synaptogenesis.

Cx45 colocalizes with Synaptophysin (Syp) shortly before eye opening in the IPL of the mouse retina, which could indicate a potential role during synaptogenesis. A recent study in the leech indicates that the interactions between chemical and electrical synapses play a critical role in synaptogenesis in invertebrates. (Baker & Macagno, 2017) show that innexins (functionally analogous to the vertebrate connexins) have an instructive role as recognition and adhesion factors during synaptogenesis in the leech. Known adhesion factors, such as neurexins, neuroligins and SynCAMs, not only connect pre‐ and postsynaptic neurons but also mediate recognition and signalling processes that are essential for synaptogenesis (Südhof, 2018). A role as adhesion or recognition factor for connexins is not yet reported for vertebrates though a handful of studies suggest that certain connexin types might, howsoever, be involved in synaptogenesis. For example, when Cx26 electrical synapses were disrupted in the developing rat neocortex, the chemical synapse and thus connections failed to form (Yu et al., 2012). In line with that, synaptogenesis of Cx36 and GABAergic connections of fast‐spiking cells takes place in the same period in the developing mouse neocortex (Pangratz‐Fuehrer & Hestrin, 2011).

This study shows that Cx45 is more likely to colocalized with Syp shortly before eye opening, indicating that it is physically located on newly formed synapses. In addition, Cx45 and Cx36 form more heterotypic junctions in both plexiform layers at that critical time. If Cx45 plays a functional role during synaptogenesis and/or helps to wire AII amacrine cells during the synaptic refinement process is debatable. However, a more systematic study is needed to determine the functional role of Cx45 for the contribution to synaptic refinement.

## Data Availability

Data available on request from the authors
